# “Talk to me, not at me”: obese women’s experiences of birth and their encounter with birth attendants—a qualitative study

**DOI:** 10.1080/17482631.2020.1845286

**Published:** 2020-11-12

**Authors:** Katrin Erna Thorbjörnsdottir, Ida Emilie Karlsen, Bente Dahl, Idun Røseth

**Affiliations:** a Center for Women’s, Family and Child Health, Faculty of Health Sciences, University of South-Eastern Norway, Kongsberg, Norway; b Department of Child and Adolescent Mental Health, Telemark Hospital Trust, Skien, Norway

**Keywords:** Experience, obesity, childbirth, birth attendants, midwives, phenomenology

## Abstract

**Purpose**: To explore the birth experiences of obese women in regard to their encounter with birth attendants.

**Methods**: Qualitative, in-depth interviews with 10 women were conducted in February 2020. Data were analysed using a descriptive phenomenological method.

**Results**: Four interrelated constituents were identified: The preconception and prejudice of being unhealthy and less able; Being unique among all the other unique women; “Talk to me, not at me”—the importance of information and communication, and; Feeling secure enough to be in the 'birthing bubble'.

**Conclusion**: For the women in our study, being obese meant experiencing challenges as well as opportunities during childbirth and in their encounter with birth attendants. Experiences of preconceptions, alienation, a focus on risk and a loss of autonomy in encounters with birth attendants were found to negatively impact the birthing process. The women desired affirmative and inclusive encounters; these kinds of encounters may improve the birth experiences of obese women.

## Introduction

According to the World Health Organization (WHO, 2020), the incidence of obesity has almost tripled in the last 45 years; in 2016, 40% of all adult women were overweight (body mass index (BMI) > 25) and 15% were obese (BMI > 30). A study conducted in 2013 reported that approximately one in five women of childbearing age in Norway were obese (Midthjell et al., 2013). Overweight and obesity therefore represent a continuing challenge in society and healthcare today.

There is an increased risk of multiple complications in pregnancy and childbirth for obese women and their children, such as preeclampsia, foetal malformations, intrauterine foetal death, prolonged birth, macrosomia, shoulder dystocia, caesarean section, maternal infection and postpartum bleeding (Marchi et al., 2015). Consequently, obese women giving birth in hospitals face additional restrictions, interventions and protocols, such as early initiation of epidural analgesia, induction of labour and continuous foetal monitoring (Magnussen et al., n.d.).

Research indicates that overweight and obese women generally struggle to receive adequate care from healthcare services and more focus is needed on tailoring resources, facilities and communication to their specific needs (Brown et al., 2006; Merrill & Grassley, 2008). Pregnant obese women have described experiences of humiliation, stigmatization, discomfort and increased stress as a result of discussing their weight with a healthcare professional (Furber & McGowan, 2011). Moreover, they may experience confusion and feel misunderstood when their need for individualized care is not met and when interventions become increasingly medicalized (Smith & Lavender, 2011).

Women are particularly vulnerable when pregnant and obese, as their larger size becomes even more visible. This can elicit negative feelings for some women, who many experience constant awareness of their body due to frequent follow-ups and extensive observation by healthcare professionals (Nyman et al., 2010). Healthcare professionals may also face challenges in relation to overweight and obese women, including unclear communication, prejudice, stigmatization and avoidance in their care provision.

Maternity services in Norway are decentralized and differentiated and take place in university hospitals/central hospitals, small and medium-sized hospitals and smaller midwife-led birth units. Home-birth is an option, but very few women choose to give birth at home. The provision of care is itself restricted by medicalized interventions and unaccommodating equipment (Heslehurst et al., 2007; Schmied et al., 2011) and the use of strict protocols and guidelines, e.g., for obese women in labour, decreases women’s opportunity for shared decision-making in labour. By categorizing large women who are otherwise healthy as “high risk”, the healthcare system may contribute to the marginalization of large women. At the same time, overweight and obese women have a higher risk of complications in labour. Some providers describe their efforts to pursue normalcy in birthing situations considered “high risk”, but report that these efforts are often unsuccessful (Singleton & Furber, 2014).

Overweight and obese women’s experiences with healthcare professionals during pregnancy is well documented, but less is known about these encounters during labour and birth (Furber & McGowan, 2011; Mulherin et al., 2013; Nyman et al., 2010). As such, the present study focused on exploring obese women’s experiences of childbirth and their encounter with birth attendants. Throughout the article the term birth attendant and healthcare personnel will encompass all skilled birth attendants involved in the birthing process, mainly midwifes, obstetricians and in some cases anaesthesiologists.

## Methods

We chose to conduct a qualitative study, following the phenomenological perspective of Husserl (1960) and Merleau-Ponty(2012). Within the phenomenological perspective, consciousness is intentional, and always directed towards something other than itself. As such, it is through one’s intentional consciousness that one constitutes, and is partly constituted by, one’s lifeworld. Phenomenology aims to describe the meanings of that lifeworld conveyed in the experiences, emotions and understandings of individuals.

A disciplined empathic stance is an important part of the phenomenological method, to ensure fidelity to the phenomenon and the “bracketing” of any preconceptions (Morley, 2010). Bracketing means to suspend, or put in brackets, all preconceptions and theories about the phenomenon. While bracketing, we adopted the disciplined empathic stance, projecting ourselves into, and thus gaining access to, others’ experiences and emotions. Admittedly, not from a first-personal perspective (Stein, 1989). Another important aspect of the phenomenological method is to withhold the existential claim—refraining from judgement concerning the reality of what is being addressed, and directing one’s attention instead towards its meaning (Husserl, 1960). We used Giorgi’s (2009) descriptive phenomenological approach to analyse data concerning the phenomenon “being obese in childbirth and their encounter with birth attendants”.

### Participants

A convenience sample was recruited via Facebook. The two first authors published a shareable post describing the inclusion criteria and contact information, and encouraged others to share this post. To be included in the study, women needed to have had a BMI above 30 at the beginning of a pregnancy in the previous five years, and to have birthed at any hospital in the southeast of Norway during that time. They were required to speak Norwegian sufficiently and be over 18 years of age. Twenty-three women contacted us: of these, 10 did not meet the inclusion criteria and 3 did not contact us again after receiving additional information. Ten women were ultimately included in the study. They were between 22 and 41 years of age, 9 had a university-level education, and their BMIs ranged from 32 to 48. They had no other comorbidities. Eight of the women were primiparous and two were multiparous. Two of the women reached near full dilatation but ended up having a caesarean section. The multiparous women gave accounts of each of their births. The births took place in hospitals of varying size in south-eastern Norway.

### Data collection and analysis

Data were collected via semi-structured face-to-face interviews with the aid of an interview guide. The disciplined emphatic stance, as described above, where employed all throughout the interviews. The interviews began with an open-ended question (“Would you tell us about your birth and your experience with encountering birth attendants?”), followed by additional questions where appropriate to facilitate a deeper exploration of the informant’s experience. The participants determined the location of the interview. Seven chose to invite us to their homes, and we met three at a café. The two first authors were present for all of the interviews but one, when only one author participated. The interviews were conducted in February 2020, with a duration of between 23 and 70 minutes. All interviews were audio-recorded and transcribed verbatim.

As detailed in the methods section, we used Giorgi’s (2009) descriptive phenomenological method to analyse the interviews and abstract the essential meanings conveyed in the participants’ descriptions. The disciplined empathic stance, bracketing and withholding the existential claim as described earlier in the method section, were exercised all through the data analysis. The interviews were read by each of the authors to provide a sense of the whole. Each interview was then sectioned into meaning units, denoting a shift in meaning relevant to the phenomenon under investigation. The next step was the transformation of the meaning units into a phenomenological sensitive language. Within this step, we used an eidetic reflection called “imaginative variation”, abstracting meaning from the specific and particular to the more general level that encompass the experience of several persons. Through this, we aimed to extract meaning which was implicitly conveyed in the concrete individual descriptions. This resulted in a description of a general meaning structure, which we separated into four essential constituents. Through this process, the data were combed through and discussed in detail among the authors to ensure transparency.

### Ethics

Approval from the Norwegian Centre for Research Data was obtained (ref. nr: 348,281). The Norwegian Regional Committee for Medical and Health Research Ethics assessed the study and concluded that it lay outside the scope of their practice (ref. nr: 94,714). The study was carried out in accordance with the Declaration of Helsinki (WMA, 2013). Participants received oral and written information and provided written informed consent for participation. They were ensured anonymity, confidentiality and the deletion of data, and were informed of their right to withdraw from the study at any time without consequence (Thagaard, 2018).

## Results

In the interviews, the participants shared their lived experiences of giving birth while being obese and their encounter with birth attendants. We identified the following general meaning structure within these experiences:

Being obese in childbirth may lead to experiences like undergoing multiple assessments and screenings on the grounds of presumed risk, despite continuous normal results. For some, this focus on risk evoked feelings of anxiety and, with the perceived bias, feelings of shame. This was experienced by the participants as being subject to prejudice and discrimination, and as if failure was expected because of their weight.

The participants craved acknowledgement and recognition of themselves as persons, but often experienced being viewed as an object, constrained by a norm that they were meant to fit but did not. This, in turn, brought forward the feeling that they were being stereotyped, as though all obese women were alike. They wanted to be considered and treated as normal birthing women, which meant being seen as persons with separate needs.

The participants experienced a lack of individualized care, a lack of information and a lack of autonomy—all of which they felt to be unjust. For all participants, it was vital to feel included in decision-making during the childbirth process and to be heard as well as seen. For some participants, coercion was experienced as an unavoidable and violating part of “the obese package” offered by some of the birth attendants (including both doctors and midwives).

Underlying this was what the participants described as a stressful and complicated relationship with their bodies, which shaped and influenced their view of themselves and how they experienced others viewing them. This underscored participants expressed need to experience a sense of security and support in the birthing room, and faith in their ability to birth, was crucial for the participants.

As part of the analysis, we have divided the above general meaning structure into four interrelated constituents: *The preconception and prejudice of being unhealthy and less able; Being unique among all the other unique women*; “*Talk to me, not at me*”*- the importance of information and communication*; and lastly; *Feeling secure enough to be in the “birthing bubble”*.

### The preconception and prejudice of being unhealthy and less able

Many of the participants experienced preconceptions and prejudice from healthcare professionals concerning their body composition and how they were perceived to live their life. One participant felt the general expectation of some healthcare professionals to be that obese people already had “one foot in the grave”. Many participants described several instances in which multiple medical tests were performed, primarily on the grounds that the healthcare professionals—mainly doctors—did not believe that the results could be within a normal range. One participant explained, “You get tested so many times, and everything was always really good—blood pressure, blood sugar … everything was always really great, so you know, it was a little rude” (i10).

Several participants experienced repeatedly being instructed on nutrition and activity. Some felt this was inappropriate, as they considered themselves to be informed about the subject and that they lived a healthy lifestyle, and thus felt shamed. Some mentioned that they tried to prevent the prejudice by being open about their situation, talking about it and asking for proper care once they became pregnant. Despite their efforts, however, they still experienced disrespect and ambivalence. One participant described the way she was greeted: “It was fake in a way, they tried to be nice, but they measure you up and down with their eyes and they just—they just see that you won’t be able to do this” (i2).

A focus on risk caused many participants to experience what they felt to be unhelpful concern. Several reported that they were already well-informed, having researched weight-specific information on pregnancy and birth, and knew the risks involved. Some described much of the information they found—from national guidelines, scientific journals and the media—as discouraging, and as supporting the notion that some would later hear from healthcare professionals: that they would not be able to carry out a successful pregnancy and birth a child without medical interventions.

Participants acknowledged that, on the one hand, they needed to be informed about obesity-related risks in pregnancy and birth. However, they also described needing to hear the positives, and to receive encouragement and support along with the facts—all of which they wanted communicated to them in a respectful and supportive manner. A few participants experienced what they perceived as an injustice when they were not allowed to birth as they wished because of hospital protocols. One participant (i10), for example, had hoped for an in-hospital waterbirth but was told that this would not be allowed because of the risks involved. She found the information and evidence with which she was provided to be lacking, and decided to thoroughly research the topic herself. She then argued for her case, and was eventually given permission to labour (and possibly birth) in water, if circumstances allowed. Other participants, however, felt a need to comply with and accept what they were told by healthcare professionals and birth attendants, and offered no opposition.

The participants described several instances in which their birth attendants expected them to have restricted mobility. One participant (i4) was informed that she was not allowed to leave the hospital bed postpartum, although she had expressed her desire to do so. The reason she was given was that the birth attendants would find it difficult to help her back up if she fell, which they feared she might. Experiencing preconceptions with regard to their fitness, health and capabilities brought forth feelings of abnormality and being less able to give birth; for some, this also evoked shame in relation to their body. One participant (i6), for example, experienced her weight as very private, and described an instance in which healthcare professionals discussed her weight in front of her partner—as though this was a subject she freely spoke about with her partner, which it was not. She experienced this as hurtful, and she felt shamed and humiliated; moreover, she found that the preconceptions and constant scrutiny experienced during this vulnerable period gave rise to feelings that had previously lain dormant.

Being subject to negative preconceptions by health professionals during pregnancy also contributed substantially to negative feelings towards their birthing self for some of the participants. For one participant, the constant focus on nutrition and activity evoked feelings of shame. She felt judged and experienced that healthcare professionals looked at her in a way that told her that she was different, abnormal and could never meet the criteria of being “normal”. She knew that their advice and information about nutrition and activity was well-intended and might not reflect the actual opinions of her carer, but she felt it still highlighted how she was different from others.

The primary care doctor, he was very preoccupied with, “If you exercise just a little … ” (…) I appreciated it a lot, he’s doing his best and does what he can and such, but at the same time, it’s … it’s that, which brings the shame forward … which makes it so that one is ashamed of oneself … and that you feel yourself completely useless, in a way, (…) and [like] one who is never going to achieve the criteria of being normal in terms of the A4 (mainstream) personality … (i3)

In contrast to the above experiences, a few of the participants reported having had singularly positive experiences in their encounter with healthcare professionals and birth attendants, in which their obesity was not the primary discussion topic. They experienced being provided with constructive information and support, and the recurrent prenatal assessments were seen as reassuring and exciting. One woman explained how she wished this would be attained:

You need to, in a way, be able to see the whole picture and be able to talk about it in a nice, non-judgmental way, in a way … For me, it’s about keeping focus on what factors actually matter (…), to be able to refer to specific studies and not just that which is preconceived, that just because you are fat that all those factors will play a role, that one, in a way, must look at the whole picture, so if one is otherwise healthy, being fat might not necessarily be such a scary factor … (i10)

She wished for healthcare personnel to see beyond the exterior of her body. To interact and communicate factually without prejudice and with respect to her, holistically, so that she too, could have a positive experience.

### Being unique among all the other unique women

I … felt she really … she really cared about us. It wasn’t just like, “Here we have yet another couple about to birth”. That … means something, that every birth is, in a way, special … (i5)

Being met as a person—feeling seen as a subject and a unique person, rather than as an object in the form of an obese body identical to all other obese bodies—was experienced as valuable and critical for most participants. Some participants felt treated as an object that was served standardized information thought to fit every pregnant and birthing person with a higher BMI. They did not experience being individually assessed or asked about their need for information or about the knowledge they already had. They experienced being a statistic, just numbers on a screen rather than a unique person—and found the experience of not being seen to be hurtful. This feeling of being overlooked as a unique person was further illustrated by some participants’ experiences of feeling discredited when machines were consulted instead of the women themselves.

Several participants talked about and emphasized how feeling normal—not standing out or feeling different—is important. As one woman stated, “when it is constantly indicated that you are overweight, you feel like something that is on the side of normal … Everyone wants to be normal, [especially] when you are fat. You absolutely don’t want to—It’s so … it’s so very tiring, to be … Well, because I know damn well that I am fat … ” (i6). Other participants talked about positive encounters with healthcare professionals and birth attendants; they described these encounters as being met as just another birthing woman, where their BMI was not a topic of discussion and where they felt seen, heard and respected as a human being. They detailed how birth attendants raised their spirits with encouragement and their presence: these participants felt special, not just one of many. One participant described this as feeling “that one is a unique woman even among all the other unique women” (i3). These participants felt recognized and approved of for who they were, and this validation and encouragement made their encounter a positive experience.

The struggle with asserting autonomy and receiving individualized care is reflected in some of the participants’ restrictions in their choice of birth place. One of the participants experienced being denied permission to birth at her local hospital because of her high BMI, despite her repeated request. She explained that the idea of birthing at her local hospital felt more secure and familiar to her, and that the refusal brought on feelings of dismissal, abnormality, confusion and anxiety. In this case, as in several others in our study, the participant experienced immense support from her primary midwife, who tried to appeal the decision on her behalf and documented incidents of a lack of individualized care. This participant described that, although the institutional decision remained unchanged, she appreciated this support, and experienced it as meaningful. Others felt their autonomy constrained by strict medical procedures.

And then it [an epidural] needs to stay in, just in case. But it is just because I’ve got a high BMI. They would never have done this to a thin human being. A normal human being. Now I feel it was a bit wrong. I am, surely, as able to birth as a normal human being. (i2)

As this quote portrays, some participants felt discriminated against, having received little support when voicing their choices, and having their freedom to choose stripped from them because they were not seen as normal birthing women. This was described as a constant struggle to fit in and fight for oneself.

### “Talk to me, not at me”—the importance of information and communication

Several participants, as mentioned earlier, talked about how they had prepared for the birth; they possessed a great deal of knowledge and expected to receive concrete and accurate information from healthcare professionals. Some, however, experienced quite the opposite, encountering a lack of information as to why particular interventions were needed. Some mentioned being told that certain interventions were necessary because of their BMI—that it was hospital protocol, to which they simply had to adhere: “One misses, maybe, some of that information … the explanation and … one becomes a little uncertain if people know why themselves … if they only do things according to the procedure” (i8). Moreover, the way in which information was communicated was important to the participants, who explained that information should be provided in a way that is understandable and makes the birthing woman and her partner feel accommodated.

The participants talked about the positive feelings engendered when healthcare personnel and birth attendants showed a genuine interest in who they were as a person and what mattered to them—that they, personally, could have an impact on what happens and that everyone involved was working together towards a mutual goal. It was described as a positive experience to be told to listen to their own intuition and body, as well as to hear that this was their body, their birth and that they were in control. The importance of mutual decision-making shines through in many of the participants’ stories. What they all have in common is the expression of wanting to feel included and involved in their own care and treatment, and to possess some degree of control over their own birth. As one of the participants responded, when asked what she wished for in regard to communication, information and respect: “Talk to me, not at me” (i4).

A few of the participants experienced having what they described as an easy connection with their birth attendant, where their wishes were respected and wants accommodated wherever and whenever possible. They highlighted how humour combined with tranquillity, effective communication, and mutual decision-making made the participants feel safe, at ease and in control. A few had experienced birthing with a familiar birth attendant, and these participants reported the greatest satisfaction out of all the participants. Moreover, the communication and interaction between the involved healthcare professionals was described as having an impact: a positive emotional tone and clear cooperation positively influenced the birth room’s atmosphere. This experience was unfortunately not universal for our participants.

Well, so, I eventually decided, fine, I’ll have the epidural. I didn’t do it because I thought I wouldn’t cope without it, but because I was a little afraid—what if something were to happen? It was kind of the fear that motivated me a little. (i7)

They said, “Because you are obese and follow this programme, we are required to insert an epidural catheter in you now … We are not required to give you the analgesics, but we need to put in the needle … Because very often one doesn’t have the stamina to complete the birth without it and that’s why it’s better to have it in place as soon as possible” … I feel that kind of sucks because … I didn’t want that needle, I was a little afraid of it. (i6)

An early intervention with epidural analgesia was raised by multiple participants as a negative experience, as the quotes above present (although some participants experienced it as a profound relief). The participants who reported negative experiences with this intervention described it as violating; many had not wanted the epidural inserted and felt it to be meaningless. The reason for the intervention was often poorly explained: some participants were told it was precautionary, in anticipation of further intervention, but most were told simply that it was hospital protocol and that they had no choice but to accept it. Some described trying to stand their ground but being coerced and not having the strength to follow through. For a few of these women, the epidural catheter was removed postpartum without ever being used. This focus on risk evoked fear and doubt in the mind of the participants, and they experienced losing faith in their own knowledge and feeling that they must comply.

### Feeling secure enough to be in the “birthing bubble”

The participants described a range of experiences concerning follow-up from healthcare professionals throughout their pregnancy. They also described how their birth pathway was laid during their pregnancy, and that their observations and encounters during this time affected how they experienced their encounter with birth attendants. Many disclosed how, throughout their lives, they had had a stressful relationship with their own body; they described this as affecting how they viewed themselves and how they perceived others viewing them. One participant conveyed how important it felt to her that birth attendants were aware of the “baggage” every birthing person brings with them. Being met by a birth attendant who possessed confidence and kindness, conveyed tranquillity and communicated information efficiently was experienced as essential and as helping to dispel anxiety. In contrast, another participant described how her own feelings of stress and uncertainty were also being reflected by the birth attendants; this affected her birthing experience negatively and made her feel unsafe. Another described how affirming it was to hear that someone saw her for exactly who she was, and that this gave her the confidence to be as she wanted to be, by believing in her strength and ability to birth. Some of the participants related how affirmation and praise made them flourish and encouraged them to rely more on themselves and their own intuition; this was experienced as freeing and relieving, and made for a positive birth experience. According to one woman, “I very much felt that I got free range to be myself and with that I was also able to have a fantastic birth experience … All that which had to do with weight and body and stomach and thighs and such things was not seen as anything special (i3).

The participants experienced letting go and not thinking about anyone else’s opinions and thoughts about their appearance to be relaxing. The birth was described as the focal point and nothing else seemed to matter, especially in the later stages of the birth. Most of the participants related how the number of healthcare professionals present in the room did not matter to them. For some, more people in the room made them feel safer; others described not minding who was present primarily because they were too preoccupied with themselves and the birthing process. The opportunity to fully occupy a space as one is, to be present and in control, was highlighted by some of the participants as a part of their positive birthing experience that strengthened, elevated and transformed them. Many of the participants described how they withdrew into themselves and disappeared into the “birthing bubble”: they did not think about their appearance nor register their surroundings, deeply concentrating only on the birthing process. This retreat into the “birthing bubble” was described as an effort to maintain control. Those who experienced being skilfully cared for explained how they knew that while they were preoccupied within the bubble, the birth attendant had control of the external setting: “You are so inside yourself also. You are in the birthing bubble … so it didn’t matter … And you feel safe then … because you know that if anything happens, there’s someone that will help you” (i9).

Experiencing an empowering and positive birth gave some of the participants a new perspective on their body and their capabilities. They experienced being strong, proud and confident, and then carried this new body image with them into other aspects of their life, both short- and long-term.

## Discussion

In this study, we aimed to explore the experiences of obese women in childbirth and in their encounter with birth attendants. The results indicate that the encounter with birth attendants can both negatively and positively affect the childbirth experience, in several ways. Despite differences in the individual experiences, there were several commonalities concerning how the encounter was experienced by the women in our study.

The first part of this article’s title—“talk to me, not at me”—is seen as capturing an important recurring description of encounters with birth attendants, in which participants experienced discrimination, objectification, inadequate information and communication, and a lack of mutual decision-making. When the participants did experience being talked to, collaborated with, supported and adequately informed, they felt met personally and emotionally during their birth, and their encounter with birth attendants was experienced as positive and fulfiling.

Lundgren and Berg’s (2007) synthesis of the midwife–woman relationship identified several key concepts essential in caring for women in both low- and high-risk pregnancies 2007, namely: surrender; trust; participation; loneliness; differentness; and creation of meaning. These concepts are all reflected in the experiences of our participants, as well. In addition, the expectations held by our participants regarding what constitutes an appropriate response from all healthcare professionals also correspond with Lundgren and Berg’s findings. We thus see parallel experiences between our obese birthing women, and women receiving healthcare in general. Our findings also coincide with Berg’s (2005) midwifery model of care for childbearing women considered high risk, where constituents of ideal midwifery care are presented.

The participants emphasized how healthcare professionals’ preconceptions about their health, fitness and abilities resulted in expectations of failure and feelings of discrimination; these, in turn, were enforced by a focus on risk. These experiences are reflected in studies addressing similar encounters, both from the perspective of women and of healthcare professionals (Bombak et al., 2016; Cook et al., 2019; Furber & McGowan, 2011; Lindhardt et al., 2013; Mulherin et al., 2013; Nyman et al., 2010). Anticipation of negative stigmatization and stereotyping has also been described in other studies, and is said to be increased by the visible nature of obesity (Brown et al., 2006; Puhl & Heuer, 2010). For our participants, this kind of negative encounters brought forward feelings of shame, humiliation and inadequacy; it also led to feelings of being different from the general norm, which has been reported by women in other studies, as well (Bombak et al., 2016; Nyman et al., 2010).

Being “normal” was described by our participants as being treated like other (non-overweight or -obese) women, not being overlooked and automatically classified based on their physical appearance and BMI. Feelings of “wanting to be normal” or “wanting to fit in” with other pregnant women have been described in other studies that have explored the experiences of obese women encountering healthcare professionals—both in general and in pregnancy and birth (Merrill & Grassley, 2008; Nyman et al., 2010). The feeling of being different is highlighted by the ill-adapted hospital environment they encounter, where blood pressure cuffs and gowns may not fit or technical equipment (e.g., foetal heartrate monitors or ultrasound machines) have limited functionality in evaluating and observing through thicker abdominal layers (Heslehurst et al., 2007; Merrill & Grassley, 2008; Nyman et al., 2010; Singleton & Furber, 2014). Our participants reported feeling that they faced scrutiny and constant reminders that they stand out. Despite the expressed desire of obese women to feel normal, some healthcare professionals have concerns about the increasing normalization of obesity, which they consider a growing challenge (Schmied et al., 2011).

According to the phenomenology of Merleau-Ponty(2012), not only is one’s access to the world gained through one’s body, but one is also conscious of one’s body through the world, or the eyes of “the other”. Merleau-Ponty described how our physical body or object (*körpe*), is different from the body as lived (Leib) through which we intentionally extend into the world. Furthermore, how a feeling of disconnection and alienation may result from treating persons as physical objects—objects that can be handled. In the women’s subjective experience, their physical body as a problematic object becomes in-focus while they, as persons or subjects, fade into the background. This sense of alienation and objectification may also restrict one’s sense of self-efficacy and ability to act in certain situations, such as when giving birth.

Some studies indicate, however, that pregnancy may increase feelings of belonging and normality, as their larger bodies becomes more accepted and obese and overweight women realize that they, too, can get pregnant and give birth (Adolfsson et al., 2013; Lingetun et al., 2017; Lundgren & Berg, 2007). Also, some midwives indicate that they try to promote normality in births considered high-risk that often are pathologized (Singleton & Furber, 2014), and doing so is encouraged by Berg’s (2005) midwifery model of care (mentioned earlier). Many of our participants who had previously felt alienated expressed a feeling of belonging and mutual respect when they were met with openness and understanding by birth attendants. Being seen as a competent and acting subject—rather than an object controlled and manoeuvred by healthcare professionals and protocols—is to feel recognized as a unique person with their own story to tell (Berg, 2005; Lundgren & Berg, 2007; Singleton & Furber, 2014; Ueland et al., 2019). In our study, however, most of the participants found this kind of individualized care to be lacking. Berg (2005), in her model of the midwife–mother relationship, identifies sensitivity and openness for the uniqueness of every woman to be of significance in midwifery caring. She emphasizes that a midwife or birth attendant should have the individual woman at the centre of their care and be present in the moment, not preoccupied with the technical and administrational aspects of birth, as some in our study experienced. A sense of security is established through the provision of appropriate information, and having the appropriate level of knowledge; effective communication skills are also needed, to provide sensitive information in an honest and non-judgemental way (Atkinson & McNamara, 2017; Berg, 2005; Lundgren & Berg, 2007; Nyman et al., 2010). As our participants described, and has been indicated in other research, trust is gained through effective communication and a belief in the woman’s ability and the normal birthing process (Lundgren & Berg, 2007). Alongside being seen and heard, mutual respect, security and trust, and positive affirmations with praise and encouragement give women space to relax and give themselves over to the birthing process—to enter the “birthing bubble” whilst trusting the birthing attendant with external control. This finding is supported by several other studies, as well (Berg, 2005; Lundgren & Berg, 2007; Olza et al., 2018).

Positive encounters, with a feeling of collaboration and mutual decision-making, have been found to be affirmative, and bring with them a feeling of trust and security (Berg, 2005; Lundgren & Berg, 2007; Nyman et al., 2010). Several of our participants, however, described experiencing a loss of autonomy and felt deprived of the freedom of choice in labour. In these situations, the participants described birth attendants as following protocols blindly, without taking the women’s previous experiences or expressed wishes into account. This was experienced as a personal violation, but it also represents a legal violation according to Norwegian patient and consumer laws: these mandate that a healthcare professional should place the patient—in this case, the birthing woman—and her wishes at the centre of all decision-making (International Confederation of Midwifery [ICM], 2014; Pasient- og Brukerrettighetsloven, 1999). The WHO emphasizes the importance of informed consent, clarification of information and freedom of choice, and that any informed choice that a woman makes should be supported (Berg, 2005; Lundgren & Berg, 2007; WHO, 2018); moreover, they recommend that women in birth should be met with empathy, compassion, encouragement and active listening. Each of these elements resonate with our participants regarding what constitutes a good encounter with the birth attendant and a positive birth experience.

Some of our participants emphasized that a positive birth experience can be an opportunity to strengthen oneself, to increase one’s faith in oneself and the capability of one’s body. Just as negative birth experiences can have lasting impacts on women, this kind of positive experience can strengthen women’s self-confidence and their trust in others—in both the short- and the long-term (Lundgren et al., 2009).

## Strengths and limitations

As part of the study methodology, steps were taken to eliminate potential author bias: namely, two authors were present at 9 out of the 10 interviews and all authors read all interviews and agreed upon all identified meanings. However, a potential for participant bias remains, as a convenience sample was recruited through social media. All the participants were ethnically Norwegian Caucasian and spoke Norwegian and of the 10 participants, 9 had higher education. Although the discrepancies in education in relation to obesity is narrowing in later years, less-educated women are two to three times more likely to be obese (Organisation for Economic Co-operation and Development [OECD], 2017). Furthermore, people with higher education can potentially be more likely to have an increased motivation to contribute to research and have more experience with reflecting on and describing their lifeworld (Thagaard, 2018). Thus the results do not specifically reflect the experiences of less-educated obese women or women of other ethnicities or minority groups; moreover, as with most qualitative studies, individual experiences from a small cohort cannot be statistically generalized to the population as a whole. The method used, however, was designed to generate more generalized meanings, and thus our study offers insight into how the lived experience of our participants encompass meanings that may be shared by others in similar situations.

## Conclusion

For participants in our study, being obese meant experiencing both challenges and opportunities in childbirth and in the encounter with birth attendants. They experienced negative preconceptions, a focus on risk, alienation and a loss of autonomy in encounters with birth attendants, which affected the birthing process. Experiencing belief in their ability to birth, and receiving individualized, non-discriminatory support and appropriate information helped the participants feel in control and capable. Shared decision-making and a respect for the unique person and their autonomy is of key importance for birthing attendants in their support of obese women and the promotion of positive birthing experiences.

## Implications for practice

To provide personalized maternity care services for obese women, their voices need to be heard and heeded. Furthermore, they should be shown the same respect, care, support and inclusion as other women. All procedures and guidelines implemented should be knowledge-based and updated, and considerations regarding autonomy and personal wishes for labour and birth should be taken into consideration. Leaders at labour wards should facilitate ethical reflections among birth attendants concerning personal and cultural preconceptions about obese birthing women. Every single woman, whether at-risk or not, should be met as a “unique birthing woman, among all the other unique birthing women”. We hope this study can contribute to increased awareness and knowledge about the care given to obese birthing women.
